# Externally triggered smart drug delivery system encapsulating idarubicin shows superior kinetics and enhances tumoral drug uptake and response

**DOI:** 10.7150/thno.55163

**Published:** 2021-03-31

**Authors:** Tao Lu, Dieter Haemmerich, Hui Liu, Ann L.B. Seynhaeve, Gerard C. van Rhoon, Adriaan B. Houtsmuller, Timo L.M. ten Hagen

**Affiliations:** 1 Laboratory Experimental Oncology, Department of Pathology, Erasmus MC, 3015GD Rotterdam, The Netherlands.; 2 Department of Pediatrics, Medical University of South Carolina, Charleston, SC 29425, USA.; 3 Erasmus MC Cancer Institute, Department of Radiation Oncology, 3015GD Rotterdam, The Netherlands.; 4 Department of Pathology, Erasmus Optical Imaging Centre, Erasmus MC, 3000CA Rotterdam, The Netherlands.

**Keywords:** smart drug delivery system, triggered release, superior release kinetics, enhanced intratumoral uptake and distribution

## Abstract

**Rationale:** Increasing the bioavailable drug level in a tumor is the key to enhance efficacy of chemotherapy. Thermosensitive smart drug delivery systems (SDDS) in combination with local hyperthermia facilitate high local drug levels, thus improving uptake in the tumor. However, inability to rapidly and efficiently absorb the locally released drug results in reduced efficacy, as well as undesired redistribution of the drug away from the tumor to the system.

**Methods:** Based on this paradigm we propose a novel approach in which we replaced doxorubicin (DXR), one of the classic drugs for nanocarrier-based delivery, with idarubicin (IDA), a hydrophobic anthracycline used solely in the free form for treatment hematologic cancers. We established a series of *in vitro* and *in vivo* experiments to in depth study the kinetics of SDDS-based delivery, drug release, intratumor biodistribution and subsequent cell uptake.

**Results:** We demonstrate that IDA is taken up over 10 times more rapidly by cancer cells than DXR *in vitro*. Similar trend is observed in *in vivo* online imaging and less drug redistribution is shown for IDA, together resulting in 4-times higher whole tumor drug uptake for IDA vs. DXR. Together his yielded an improved intratumoral drug distribution for IDA-SDDS, translating into superior tumor response compared to DXR-SDDS treatment at the same dose. Thus, IDA - a drug that is not used for treatment of solid cancers - shows superior therapeutic index and better outcome when administered in externally triggered SDDS.

**Conclusions:** We show that a shift in selection of chemotherapeutics is urgently needed, away from the classic drugs towards selection based on properties of a chemotherapeutic in context of the nanoparticle and delivery mode, to maximize the therapeutic efficacy.

## Introduction

Tumor response is largely determined by the ability to deliver sufficient drug levels at the target site 1, 2. Conventional chemotherapy relies on systemic administration of cytotoxic agents which are transported by the bloodstream to a tumor site. This non-selective delivery of drugs leads to marginal accumulation in the tumor and dose-limiting side-effects, which cause failure of therapy and morbidity 3. Encapsulation of chemotherapeutics in nano-carriers provides a possibility to increase drug levels in tumor while diminishing side-effects, but has so far limited impact on efficacy 4-8. The disappointing efficacy of classic drug delivery carriers can be attributed to two main reasons: limited accumulation in tumor and slow drug release from the carrier. Currently approved nano-carriers depend largely on the enhanced permeability and retention (EPR) effect of solid tumors 9, 10. Importantly, the existence of an EPR effect, and thus enhanced accumulation particularly in human tumors, is under debate 11-15. Moreover, we and others show that nano-carriers are relatively stable even when taken up by tumor cells, resulting in inadequate levels of bioavailable drug 14, 16, 17.

Smart drug delivery systems (SDDS), therefore, have been developed where content release can be activated by an external trigger, enabling delivery of free drug at a high concentration to a relatively small tissue volume and in short time-frame 18, 19. Because of the versatile nature of lipid-based SDDS, clinical applicability, and high compatibility, thermosensitive liposomes are the most advanced 20-23. These SDDS are able to generate rapid release at the phase transition temperature (Tm), which induces phase separation in the liposome membrane, facilitating content release 24. Hence, when the thermosensitive SDDS enter a heated tumor (e.g. at 42 °C), the encapsulated drug is rapidly and massively released, resulting in high local drug concentrations and promoting subsequent drug diffusion and thus uptake by tumor cells 25, 26. Optimal release at around 42 °C is preferred for clinical practice as this temperature is different enough from the body temperature to allow specific heat-induced release, but without causing damage to surrounding tissues or impairing blood flow. At body temperature these SDDS are relative stable and encapsulated drug is retained during circulation 24, 27.

Doxorubicin (DXR) has been thus far the preferred drug for encapsulation in thermosensitive nano-carriers. With these systems, impressive drug levels have been achieved locally in particular when used in a so-called intravascular release setting 22, 25, 28, 29. However, this massive local-release changed the playing field as now local drug concentrations are that high that the tumor cells cannot take up the drug fast enough and redistribution of unbound drug to the body occurs 25. To enable maximum performance of externally-triggered SDDS-mediated drug delivery, the released drug should almost instantly enter tumor cells and should be retained to prevent washout. At present, encapsulated drugs in nano-carriers are typically selected based on clinical efficacy for a particular tumor type, for which these drugs are commonly used in the free form. We propose, and this is a novel way of thinking for SDDS-mediated delivery, that the chemotherapeutics which are ideal for loco-regional treatment of cancer, should be considered. Based on this paradigm we selected idarubicin (IDA), a more hydrophobic anthracycline. Free IDA shows strong protein binding affinity and short half-life in bloodstream, which limits drug delivering *in vivo* 30, 31. Therefore, IDA is used for hematologic cancers but little effectiveness is observed in solid tumor treatment 32. To improve delivery of IDA, this study established DPPC and DSPC phospholipid-based thermosensitive nano-carriers. Here, we in depth compared IDA-SDDS and DXR-SDDS drug release, cellular uptake, intratumoral distribution and antitumor activity. We show that rather than focusing on changing the materials forming the SDDS, the content of these SDDS needs to be evaluated in relationship with the functionality and application of the SDDS. Our results indicate that selection of Idarubicin for delivery by heat-triggered SDDS may improve clinical outcome in patients currently considered for treatment with DXR-based SDDS and hyperthermia.

## Methods

### Materials

1,2-distearoyl-sn-glycero-3-phosphocholine (DSPC), 1,2-dipalmitoyl-sn-glycero-3-phosphocholine (DPPC) and 1,2-distearoyl-sn-glycero-3-phosphoethanolamine-N-(amino(polyethylene glycol)-2000) (DSPE-PEG_2000_) were purchased from Lipoid (Ludwigshafen, Germany). Doxorubicin-hydrochloride solution (2 mg/mL) was purchased from Accord Healthcare. Idarubicin-hydrochloride powder was purchased from Sigma Aldrich. 4-(2-hydroxyethyl)-1-piperazineethanesulfonic acid (HEPES), (NH_4_)_2_SO_4_, (NH_4_)_2_EDTA, DMEM culture medium, fetal bovine serum (FBS), sulforhodamine B (SRB), 2-Amino-2-hydroxymethyl-propane-1,3-diol (Tris), NaCl, glycerol, Mayer's hematoxylin, and eosin Y were from Sigma Aldrich. Penicillin-streptomycin (Pen-Strep) solution was from Lonza (Breda, Netherlands). PD-10 desalting columns were from GE Healthcare Life Sciences (Buckinghamshire, UK).

### Cytotoxicity assay

Murine B16BL6 melanoma, human BLM melanoma and murine BFS-1 sarcoma cells were cultured in DMEM medium with 10% FCS. Cells were seeded in 96-well-plates at a density of 5000/well for 24 h in order to adhere and enter the exponential growth phase. Medium was removed and new medium with free IDA or DXR was added followed by incubation for 1 h or 24 h, after which drug-containing medium was replaced with new medium, followed by additional incubation for 48 h or 24 h at 37 °C. Cells were fixed using 10% (w:v) trichloroacetic acid (TCA), rinsed with water and stained with 0.5% sulforhodamine B (SRB) for 20 min. Thereafter, cells were washed with 1% acetic acid and left to dry. Thereafter 10 mM Tris was added to dissolve the SRB and measured at 510 nm. Cell survival as percentage of control is presented and IC_50_ values were calculated.

### Cell uptake

A total of 7 × 10^6^ cells were seeded in T75 flask and incubated overnight. Medium was removed and 5 ml of IDA or DXR containing medium (10 µM) was added followed by incubation for 5 min, 10 min, 30 min, 1 h, 2 h, 4 h, 8 h, 16 h and 24 h. Cells were collected and drug was extracted with 85% isopropanol (containing 0.75 M hydrochloride acid) for at least 24 h at 4 °C.

### Cellular retention

A total of 7 × 10^6^ cells were seeded in T75 flask and incubated overnight. Medium was removed and 5 ml new medium containing IDA or DXR at a concentration of 10 µM was added for 4 h. Medium was replaced by new medium followed by incubation for 1 h, 4 h, 8 h, 16 h and 24 h, after which drugs were extracted from cells with acidic isopropanol as mentioned above.

### IDA-SDDS and DXR-SDDS preparation

SDDS were composed of the phospholipids DPPC/DSPC/DSPE-PEG (60/35/5 for IDA, 70/25/5 for DXR; molar ratio) and prepared by thin lipid film hydration method, followed by heated extrusion and remote-loaded as described previously 22, 33. Briefly, 100 µmol of lipids was dissolved in methanol/chloroform (1/9 v/v), followed by evaporation under vacuum and nitrogen flush. The lipid film was hydrated in ammonium EDTA buffer (250 mM, pH 5.5 for IDA loading) or in ammonium sulfate buffer (250 mM, pH 5.5 for DXR loading). Small unilamellar liposomes were obtained by extrusion using Nuclepore® (Whatman Inc., USA) filters with pore sizes from 200 to 50 nm. A PD-10 column was used to replace the external buffer to create an ion gradient between internal (ammonium buffer, pH 5.5) and external (HEPES buffer, pH 8.5) SDDS for the so-called remote drug loading. Drug molecules are able to cross the lipid membrane, followed by protonation and formation of precipitate with EDTA or sulfate in the internal buffer 33-35. According to the optimized loading methods published previously 22, 33, IDA was encapsulated at a molar drug/lipid ration of 0.3/1, at 33 °C for 1 h, and DXR was encapsulated at a molar drug/lipid ratio of 0.15/1, at 39 °C for 1 h. Using these conditions a loading efficiency of 100% was reached for both. Drug loaded SDDS were collected by ultracentrifugation at 40,000 rpm for 2 h at 4 °C, following by resuspension in HEPES buffer (pH 6.5) overnight at 4 °C. SDDS quality was confirmed by measuring diameter and polydispersity index before and after drug loading (Table S1), and before *in vitro* and *in vivo* application.

### Cryo-TEM images of IDA-/DXR-SDDS

IDA-SDDS, DXR-SDDS and empty SDDS were examined by cryo-transmission electron microscopy (cryo-TEM) imaging with a Fei Tecnai F30ST microscope (Philips, The Netherlands) as described previously 33. Briefly, 3 μL of SDDS suspension was dropped on a lacy carbon film and subsequently snap-frozen in liquid ethane by a Vitrobot. An amorphous ice film was created, containing particles of interest.

### *In vitro* release of IDA-SDDS and DXR-SDDS

Fifty µL of 8 mM (lipids) SDDS suspension was instantly added to 3 mL 100% fetal calf serum (FCS) at 37 or 42 °C for 1 h. Real-time release of content was detected by spectrofluorometry (IDA: Ex. 485 nm/Em. 571 nm; DXR: Ex. 482 nm/Em. 594 nm) (Hitachi F-4500 Fluorescence Spectrophotometer, Japan). The average fluorescence intensity of the initial 5 seconds at 37 °C was recorded as *I_0_*, while *I_t_* represent fluorescence recorded at time points after that. After 1 h, detergent (10% Triton X-100) was used to disrupt all SDDS to measure maximal drug fluorescence, which was designated *I_max_.* Release was calculated as Release (%) = (*I_t_* - *I_0_*)/(*I_max_* - *I_0_*) × 100 as previously described 33. The same procedures were performed with IDA- or DXR-SDDS for 5 min to measure IDA and DXR release at different temperatures from 37 °C to 45 °C.

### Real-time imaging of drug release from IDA-SDDS and DXR-SDDS and subsequent uptake by tumor cells

SDDS release and uptake by tumor cells was performed using confocal imaging of Attofluor® Cell Chambers (ThermoFisher) as previously described 36. Briefly, a coverslip (25 mm in diameter) was inserted into an Attofluor chamber and subsequently sterilized. Coverslips were coated with gelatin (1 mg/mL) prior to cell seeding. B16BL6 or BLM cells (10^4^ per chamber) were seeded and incubated overnight at 37 °C. IDA- and DXR-SDDS were diluted to 10 µM using cell medium and 1 ml was added into the cell chamber after which cell chambers were placed in a temperature controlled confocal microscope. Cells were incubated at 37 °C for 2 h, followed by 10 min or 1 h at 42 °C. Similarly, free IDA or DXR (10 µM) was used to incubate with cells for 42 °C 1 h. A Zeiss LSM 510 META confocal microscope (543 nm helium-neon laser) equipped with a cell culture system was used to capture images with a 40 × (NA 1.3) oil immersion lens and analyzed using Fiji Image J.

### Local hyperthermia application on mouse study

All animal experiments were carried out in accordance with protocols approved by the committee on Animal Research of the Erasmus Medical Center (Rotterdam, the Netherlands) and in accordance with the European directive 2010/63/eu on the protection of animals used for scientific purposes. In this work, we have two settings for local hyperthermia. (I): Water bath mild hyperthermia was used for mice with tumors implanted on the lower right leg, which was submerged in heated water (42.5 °C). Non tumor tissue was protected with Vaseline. Using this setting a temperature of 41 to 42 °C is reached in the tumor. The heating lasted for 1 h in pharmacokinetics, biodistribution and efficacy studies. (II): A heating coil was used for intravital imaging. An external circular conductive heating coil was attached to the glass at the back side of the window chamber to provide homogeneous hyperthermia in the chamber area 22. Thermocouples (point-welded thin manganese and constantan wires from Thesso®, Amsterdam) were used to online monitoring the temperature.

### Plasma pharmacokinetics and biodistribution of IDA- and DXR-SDDS

A tumor piece (~3 mm^3^) of BLM melanoma was subcutaneously transplanted to the right hind leg of NMRI nu/nu mice. IDA-SDDS and DXR-SDDS were injected at either efficacy dosage (2.7 µmol/kg for IDA and 9 µmol/kg for DXR, which are the doses at which IDA and DXR respectively cause a significant and comparable tumor response, or doses as indicated below, when tumor sizes reaches ~200 mm^3^. For the first timeline blood samples were drawn at 5 min, 30 min, 1 h, 2 h, 3 h, 6 h, 12 h and 24 h, in which efficacy dose injection was applied. For the second timeline, tumor-bearing mice were kept under anesthesia and applied with local HT (42 °C) or NT (37 °C) of the tumor, injected and kept for 60 min at the set temperatures, followed by 30 min at 37 °C. Blood samples were collected at 5 min and 90 min post injection, mixed with phosphate buffered saline and Triton at a volume ratio of 30: 70: 100, followed by measurement at 485 nm excitation and 590 nm emission (Wallac 1420 Victor microplate reader). Half-life (t_1/2_) was calculated through WinNonlin analysis.

Organs and tumors were isolated from mice at times indicated, snap frozen in liquid nitrogen and stored at -80 °C until further analysis. Extraction of drugs from tissues was performed according to Laginha *et al.* 37. Briefly, all tissues were weighed and homogenized (Bio-Gen PRO200 Homogenizer) in 85% isopropanol (containing 0.75 M hydrochloride acid), then placed at 4 °C for at least 24 h for drug extraction. IDA and DXR levels were determined as described above.

### Intravital imaging of IDA and DXR release and intratumoral distribution

Installment of the dorsal skinfold window chamber and intravital imaging was performed as described previously 33, 38. C56BL/6 mice constitutively expressing an eNOS-Tag-GFP fusion protein in endothelial cells were installed with a skinfold window chamber, a B16BL6 melanoma was implanted in the chamber and imaged using intravital microscopy (Zeiss LSM 510 META). Equal doses of IDA- or DXR-SDDS (18 µmol/kg) were injected intravenously. Window chamber tumors were exposed to local hyperthermia of 42 °C, which took around 10 min to reach target temperature and were kept at 42 °C for 1 h, followed by cooling down to 37 °C for 30 min. Images was captured every ten seconds at 10 × (NA 0.3) using a Helium-Neon laser (543 nm for IDA/DXR monitoring with long pass 585 nm filter) and an argon laser (488 nm for GFP endothelial cells monitoring with band pass 505-550 nm filter). Drug fluorescence intensity was quantified by Fiji Image J and Matlab.

### Therapeutic efficacy of IDA- or DXR-SDDS

A tumor piece (~3 mm^3^) of BLM melanoma or BFS-1 sarcoma was subcutaneously transplanted within the right hind leg of NMRI nu/nu mice. When tumors reached around 100 mm^3^ in size, tumors were heated by local hyperthermia of 42 °C (HT) or normothemia (37 °C, NT), directly after a single i.v. injection of IDA- (2.7 µmol/kg) or DXR- (9 µmol/kg) SDDS, for 1 h as previously reported 22, 33. Similarly, single dosage of free IDA (2.7 µmol/kg) or DXR (9 µmol/kg) was intravenously injected in mice followed by local hyperthermia of 42 °C 1 h as a comparison. Weight and tumor growth was recorded daily and tumor volume was calculated using the formula Length × Width × Depth × 0.4. Mice were euthanized when tumor reached ~15 × 15 × 15 mm^3^ in size or based on human endpoints.

### Histology

After treatment, tumors were excised, fixed in 25% (v/v) paraformaldehyde for 24 h, followed by paraffin embedding, and 5 µm slices were cut and stained with hematoxylin and eosin (H&E). A second set of tumors were excised from eNOS-Tag-GFP mice, snap frozen, cut into 5 µm frozen slices and imaged by confocal microscopy to study intratumor distribution.

### Statistics

Results are analyzed using Mann-Whitney U test and presented as mean ± SD or mean ± SEM. *p* values below 0.05 were considered significant.

## Results

### Free IDA shows faster cellular uptake and longer retention than DXR

**Figure 1:**
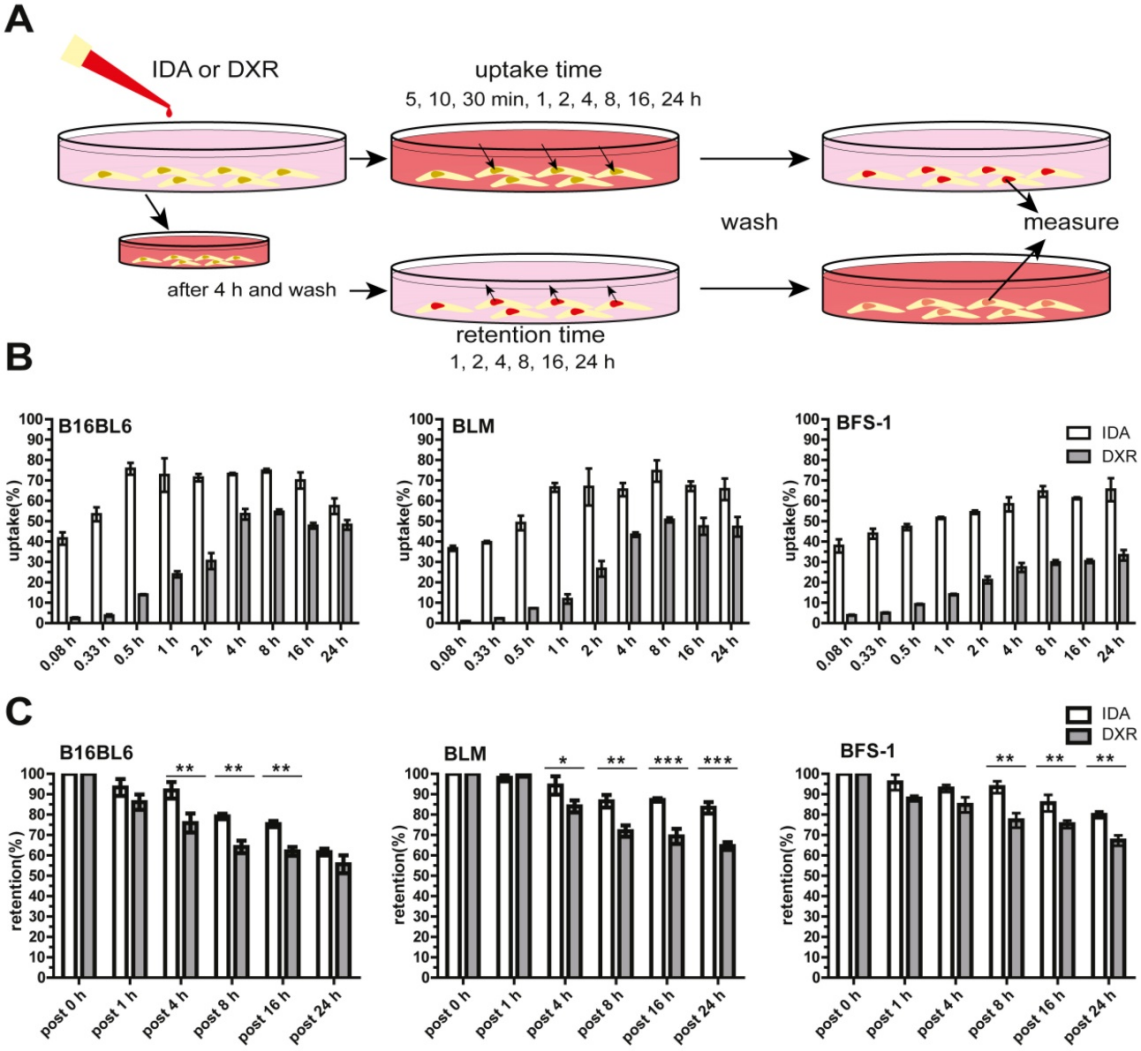
** Faster and higher idarubicin (IDA) uptake by tumor cells *in vitro* compared to doxorubicin (DXR). (A)** Schematic illustration of uptake and retention determination *in vitro*. **(B)** B16BL6 (left), BLM (middle) and BFS-1 (right) cells display faster uptake of free IDA than DXR (n = 4 per cell line). **(C)** Extended cellular retention of free IDA is observed compared to DXR (n = 3 per cell line, Nonparametric Mann-Whitney test: *p < 0.05, **p < 0.03, ***p < 0.01). Data are presented as mean ± SD.

To assess the cellular uptake and retention of IDA and DXR, different types of tumor cells were used and exposed to the free drug at the same molar concentration (Figure 1A). We show that IDA uptake reached approximately 40% of the available drug in 5 min, followed by saturation within 0.5-1 h. While DXR accumulation was marginal during the first half hour and reached maximum after around 4 h (Figure 1B, Figure S1A). An overall 16-fold faster cellular uptake was observed for IDA compared to DXR (Figure S1B). Besides, a significantly prolonged retention of IDA in tumor cells was observed compared to DXR (Figure 1C). The rapid uptake and longer retention of IDA by cells may, at least partly, contribute to the higher cytotoxicity in comparison with DXR, showing 5-25 times higher IC_50_ value (Table 1, Figure S1C). (Encapsulation of IDA or DXR in SDDS was observed with significantly declined cytotoxicity in cells without hyperthermia in previous work by us 22, 33). Together these results suggest that idarubicin could be a better candidate for local-release delivery systems.

### *In vitro* IDA-SDDS shows faster release than DXR-SDDS and exhibits higher cellular uptake

**Figure 2:**
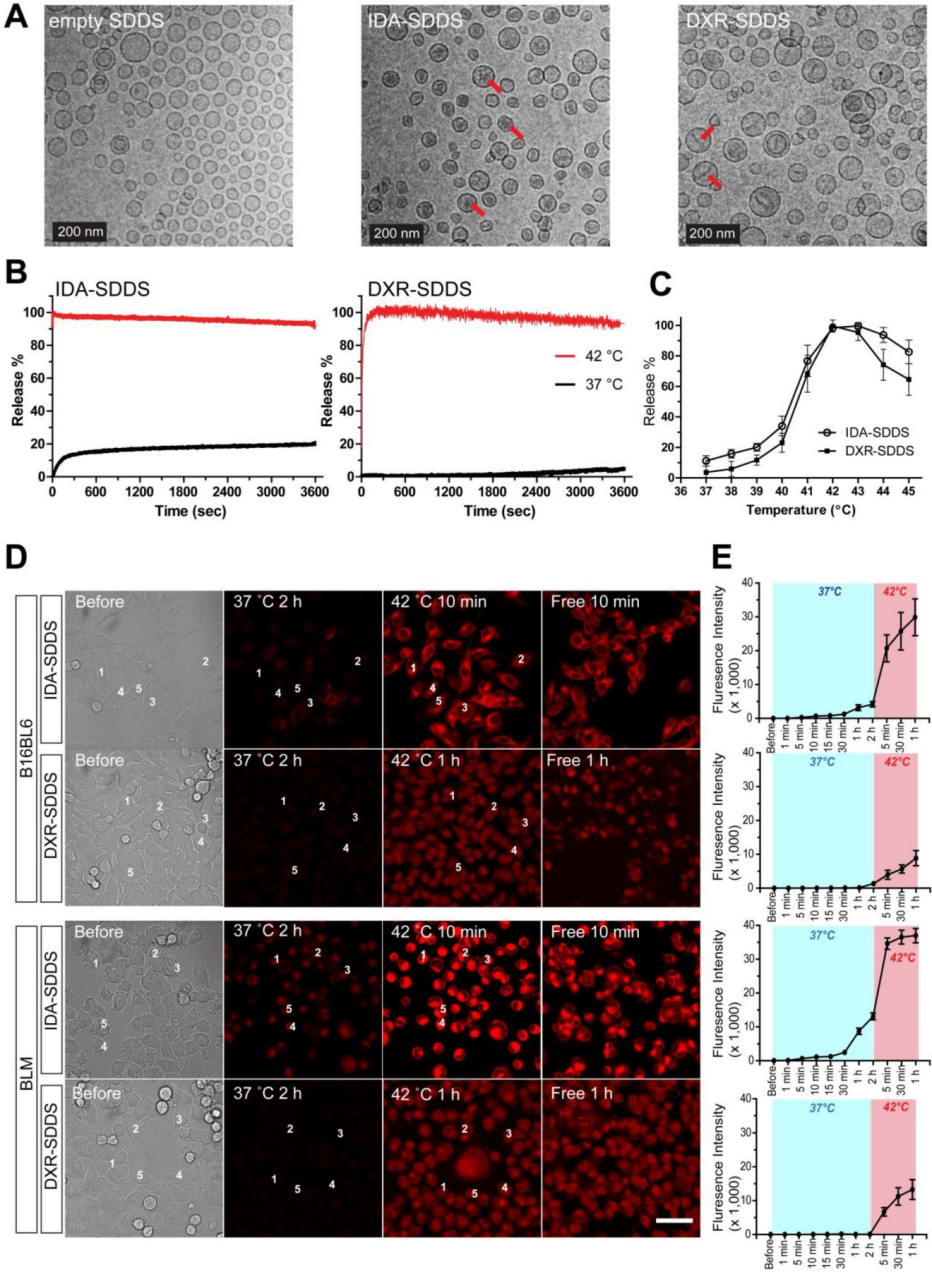
** IDA-SDDS exhibit faster release kinetics when exposed to 42 °C hyperthermia (HT) compared to DXR-SDDS. (A)** TEM photos of empty SDDS (left), IDA-SDDS (middle) and DXR-SDDS (right), show difference in crystal formation after loading (red arrows). IDA-SDDS *in vitro* time-dependent **(B)** and temperature-dependent release **(C)** in full calf serum is compared to DXR-SDDS (n = 3). **(D)** On-line confocal microscopy images of IDA and DXR uptake by B16BL6 (upper) and BLM (lower) after release from SDDS when exposed for 2 h to 37 °C followed by 1 h at 42°C. Settings: gain = 600, resolution = 512 × 512. Scale bar, 50 µm. **(E)** Five cells are randomly selected in **(D)** to track accumulation of IDA or DXR, upon release from SDDS, in tumor cells in time. Data are presented as fluorescent intensity of these 5 cells.

Delivery of bioavailable, (i.e. free, released) drug in the intravascular release setting is dependent on the amount of SDDS-encapsulated drug passing through the heated region, as well as the time these SDDS are exposed to 42 °C. Release kinetics need therefore to be fast. Here IDA- and DXR-SDDS were prepared using different phospholipid ratios to obtain fastest release at 42 °C and best stability at 37 °C (Figure 2A, Table S1). We observed *in vitro* that 100% of the IDA was released in response to 42 °C within a matter of seconds, which is desired for intravascular release-based therapy, and is faster than complete DXR release that took around 2 min (Figure 2B). Around 20% of IDA was leaked at body temperature and physiologic pH for 1 h, however, consistent with our previous observation 33, which is likely attributed to the part of IDA present in the liposomal bilayer, as prolonged incubation did not cause further leakage. In the temperature-dependent release assay, both IDA- and DXR-SDDS showed maximal release at 42-43 °C (Tm), while limited release was observed at suboptimal temperatures (Figure 2C), and size of SDDS was not affected (data not shown). Confocal imaging of live cells confirmed fast release and sequential uptake by cells of IDA, when released from SDDS after exposed to 42 °C, at a level 4-fold higher compared to DXR (Figure 2D-E). Interestingly, IDA locates predominantly in the cytoplasm 33, 39, while DXR transfers mostly to the nucleus (Figure S2).

### IDA is efficiently released from SDDS, showing increased tumor uptake compared to DXR

**Figure 3:**
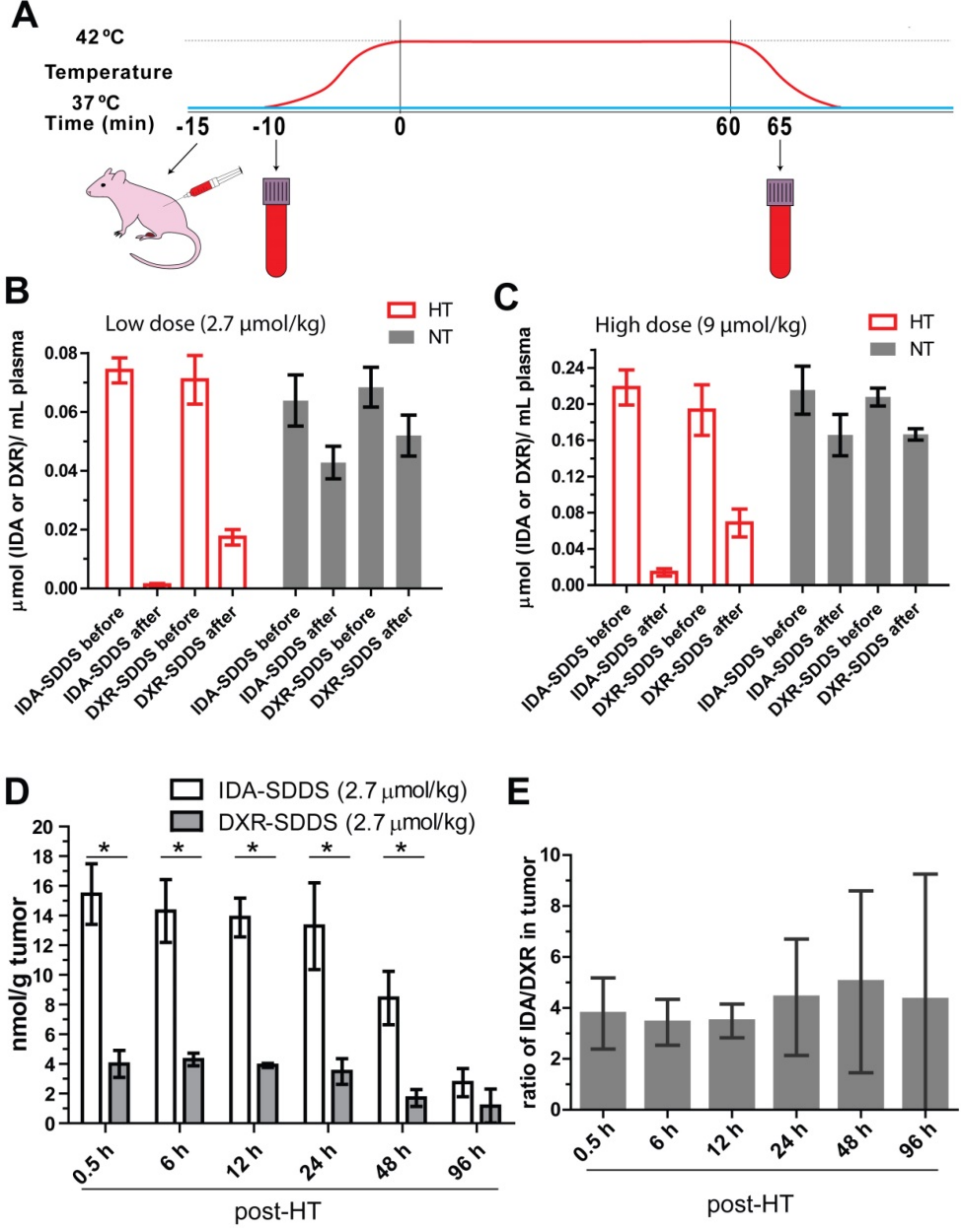
** IDA-SDDS show higher efficiency of content release and tumor uptake *in vivo* than DXR-SDDS under HT. (A)** Schematic illustration of hyperthermia treatment and collection of blood samples in mice. **(B, C)** Plasma drug concentration comparisons before and after 1 h local hyperthermia (HT) (42 °C) or normothermia (NT) (37 °C) in BFS-1 tumor bearing mice treated with low dose (A) or high dose (B) SDDS (n = 3 mice per group). Complete release is observed with IDA-SDDS plus HT. **(D)** Under HT, tumors take up released IDA more efficiently and maintained a higher drug level during 48 h post treatment compared to DXR (n ≥ 3 mice per group, Nonparametric Mann-Whitney test: *p < 0.03). **(E)** The ratio of tumor drug concentration between IDA and DXR calculated from (D) gradually increases within 48 h after treatment, confirming longer retention of IDA in cell after uptake. Data are represented as mean ± SEM.

To test release efficiency *in vivo*, tumor-bearing mice were exposed to HT of the tumor alone and injected systemically with IDA- or DXR-SDDS (Figure 3A). We observed almost complete depletion of IDA from circulation 1 h after injection of 2.7 µmol/kg IDA-SDDS in HT treated mice, indicating that IDA-SDDS that have passed the heated region almost completely released IDA (Figure 3B). Noteworthy is to consider that a considerable part of injected SDDS will be taken up by cells of the RES, and will never pass the tumor. In contrast, after 1 h of HT still 24% of injected DXR was present in the circulation indicating a less complete release from SDDS. On the other hand, in mice in which tumors were kept at 37 °C most drug remained circulating, and thus encapsulated. Comparable results for both IDA and DXR were obtained also at the higher dose of 9 µmol/kg (Figure 3C). In addition, accumulation of released IDA in tumors 30 min after 1 h HT reached 15.4 ± 3.5 nmol per gram tumor, 4-times higher than DXR which was 4.5 ± 0.5 nmol/g tumor (Figure 3D). We also observed more IDA distributed in spleen and lung after HT compared to DXR-SDDS, yet no side-effect in IDA-SDDS-treated mice was present (Figure S3), also mice showed normal behavior and tissues showed no obvious signs of toxicity. Similarly, significantly increased tumor accumulation of IDA was observed also when a high dose of 9 µmol/kg was administered (Table 2). Heterogeneity in accumulation likely results from heterogeneous makeup of the tumor with respect to vasculature, perfusion and tissue density. Overall we observed a 3.8 to 4.8-fold higher accumulation of IDA compared to DXR within the first 48 h when delivered by SDDS in HT treated mice, which is in agreement with faster uptake and longer retention of IDA observed *in vitro* (Figure 3E).

### Online intravital imaging illustrates improved IDA-SDDS release and distribution in tumor compared to DXR-SDDS

**Figure 4:**
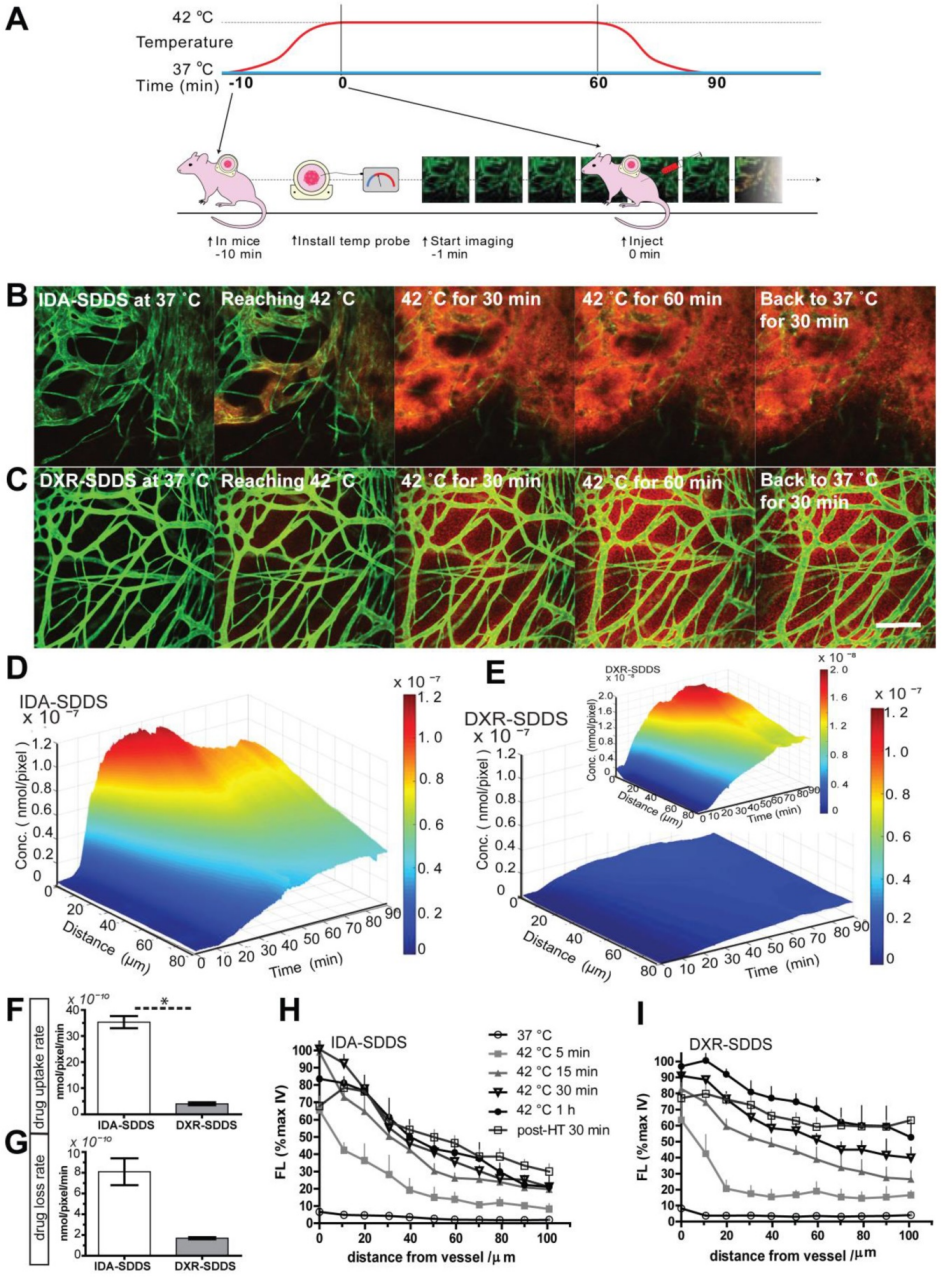
** Rapid release from SDDS and uptake by tumor cells of idarubicin (IDA) results in high local drug levels and a steep intratumoral gradient compared to doxorubicin (DXR). (A)** Schematic illustration of intravital microcopy of mice undergoing hyperthermia in combination with SDDS. **(B, C)** Imaging of heat-triggered release in a window chamber fixed on eNOS-Tag-GFP mice showing green vessels. Eighteen µmol/kg of IDA-SDDS (B) or DXR-SDDS (C) was injected followed by 1 h local hyperthermia and 30 min normothermia, showing intravascular release of drug (red) and the subsequent drug diffusion into interstitial space of tumor. Scale bar, 200 µm. **(D, E)** A 3-dimensional representation of intratumoral drug concentration, as a factor of time and penetration distance into tumor tissue from the nearest vessel, shows higher tumor uptake of IDA (D) than DXR (E) during the treatment course (insert represents DXR concentration at a smaller scale). Maximum uptake was reached earlier for IDA showing higher drug accumulation close to tumor vessels compared to DXR (n = 3 mice per group). (F) Online IDA and DXR uptake rates are calculated starting from 10 to 30 min during HT, after which IDA uptake enters a saturation state (n = 3 mice per group, Nonparametric Mann-Whitney test: *p < 0.01). **(F)** Drug efflux is calculated from the peak concentration to the beginning of the steady concentration. IDA shows around 15 min of drug efflux after which a steady concentration was maintained. **(H, I)** At specified time points, release and diffusion profiles of IDA-SDDS (H) and DXR-SDDS are depicted (I). Data are presented as mean ± SD.

While total drug accumulation may provide some indication of possible outcome, another important aspect is intratumoral distribution 14, 40. We applied intravital microscopy to gain insight in intratumoral drug distribution kinetics and profile in real time (Figure 4A). We observed accelerated accumulation of IDA, preferentially around tumor-associated vessels with IDA-SDDS in HT treated mice compared to DXR-SDDS (Figure 4B-C; Video S1, S2). Immediately upon reaching the target temperature IDA uptake in tumor cells is apparent; accumulation in cells results in more clearly visible fluorescence. As uptake of DXR by tumor cells is slower, accumulation, and thus visibility, is delayed. After around 35 min of treatment maximum IDA uptake of 6.0 × 10^-8^ nmol/pixel was reached at 60 µm from the nearest vessel (Figure 4D). In contrast, only after 50 min maximum uptake of DXR was reached with a concentration of 0.7 × 10^-8^ nmol/pixel at 60 µm (Figure 4E). Together, this results likely in a higher degree of DXR dilution and a lower drug concentration compared to IDA, and thus possibly leading to insufficient cell kill. Detailed analysis of drug uptake rate and drug loss kinetics show a 10-fold faster uptake of IDA compared to DXR before reaching saturation, indicating the high take-up efficiency of released IDA by tumor cells considering the short time of SDDS passing through the heated region (Figure 4F). Redistribution of IDA seems to occur for a period of about 15 min before reaching a steady state, showing a 12.6% loss compared to 17.0% of DXR. However, likely due to difference in absolute drug levels a 4-fold faster loss of IDA than DXR was observed (Figure 4G). Profile analysis of temporal intratumoral drug distribution confirms these observations indicating that maximum uptake is reached earlier with IDA as compared to DXR. Interestingly, IDA, likely due to the higher hydrophobicity, shows a trend of less homogeneous distribution away from vessels over time compared to DXR (Figure 4H-I).

### IDA-SDDS improves intratumoral distribution and drug concentration, enhancing therapeutic efficacy

**Figure 5:**
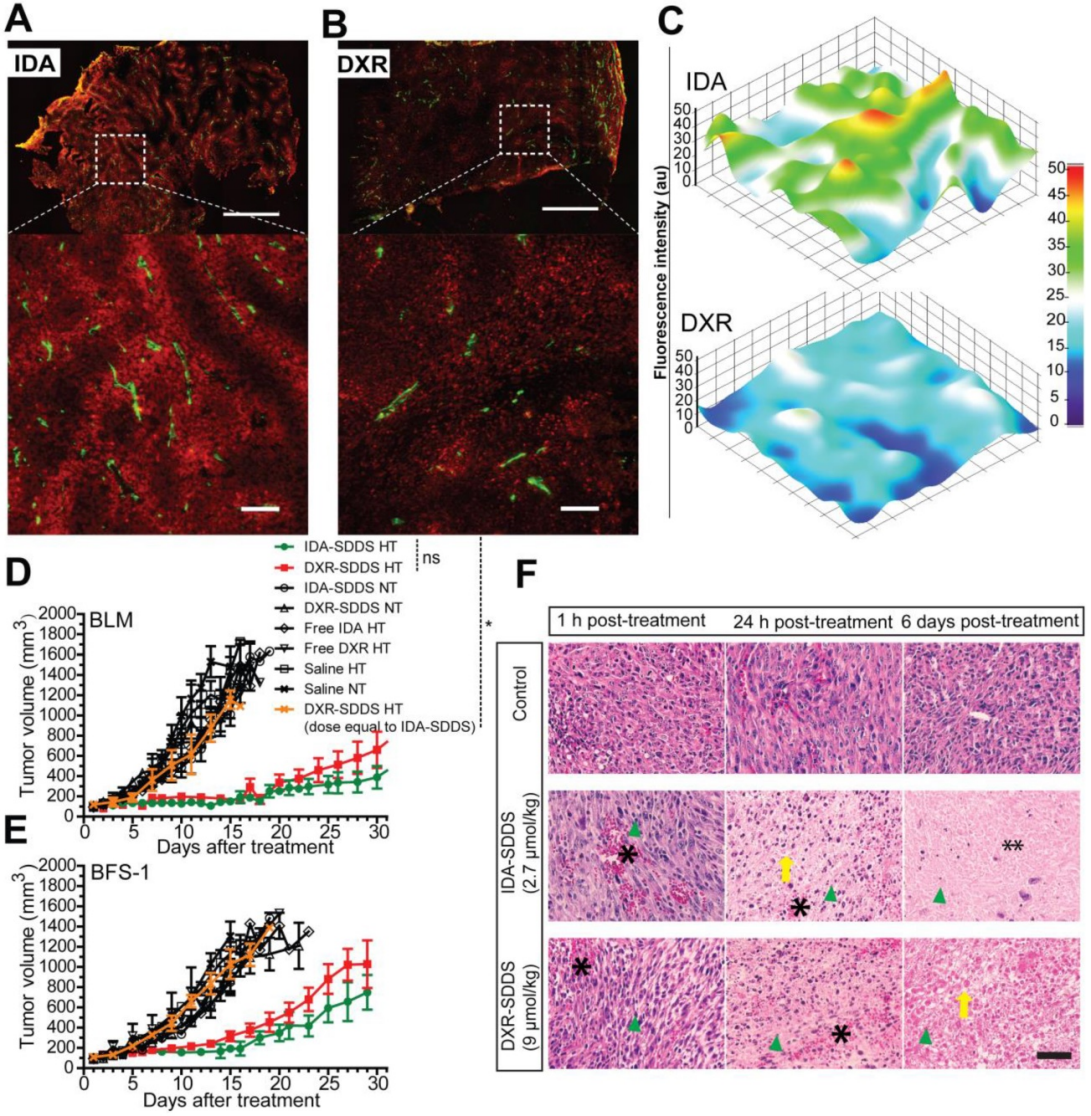
** Superior antitumor activity of IDA-SDDS in combination with hyperthermia, compared to DXR-SDDS. (A, B)** Histological analysis of eNOS-Tag-GFP mice bearing BFS-1 tumor treated with IDA-/DXR-SDDS combined with HT demonstrate higher perivascular accumulation of IDA (A) and a lower, but more uniform penetration for DXR (B). Top panel shows complete cross section and bottom panel a region at higher magnification (n = 2 mice per group). Settings: gain = 800, resolution = 1024 × 1024. Scale bars, 1 mm (top), 50 µm (bottom). **(C)** Fluorescence measurement of tumor sections confirms higher and steep perivascular gradient distribution of IDA compared to DXR. **(D, E)** Tumor response in BLM (D) and BFS-1 (E) bearing mice treated with IDA-SDDS (2.7 µmol/kg, green), DXR-SDDS (2.7 µmol/kg, yellow), or DXR-SDDS (9 µmol/kg, red) combined with hyperthermia, or normothermia. Only IDA-SDDS, or DXR-SDDS at a high dose, inflicted a tumor response. When DXR-SDDS at 2.7 µmol/kg, or DOXIL at an accumulated dose of 13.8 µmol/kg (4 injections with an interval of 4 days) were administered, all mice showed progressive disease (n = 7 each group for IDA- or DXR-SDDS HT group, n = 5 each group for the rest; Nonparametric Mann-Whitney test. *p < 0.01; ns, not significant). Data are represented as mean ± SEM. **(F)** Fixed H&E stained tumor sections at 1 h, 24 h and 6 days post-treatment with IDA- or DXR-SDDS with HT. After 24 h post-treatment, necrosis of tumor was observed, showing destruction of tumor cells (disappearance of cell structure and nuclei (nuclei indicated by green arrow head)) and vasculature (occurrence of hemorrhage (asterisk) and edema) and massive coagulative necrosis (especially in the IDA group (double asterisk)) at 6 days post treatment with IDA or DXR-SDDS plus HT, compared to normothermic controls. Scale bars, 50 µm.

Homogeneous distribution is preferred if levels are sufficiently high to kill all tumor cells. High accumulation around vessels however may not only kill tumor cells but also tumor-associated vasculature and therefore inflict a strong tumor response 41. In agreement with the intravital data, the histological study of tumor cross-sections show that high levels of intracellular IDA were present around tumor associated vessels at 30 min after 1 h HT treatment with IDA-SDDS (Fig. 5A), resulting in a steep concentration gradient away from the vessel (Figure 5C). DXR distribution was more homogeneous, but lower, with a more gradual decline into tumor tissue (Figure 5B-C). Consequently, in efficacy studies we observed that only a single injection of IDA-SDDS at 2.7 µmol/kg inflicted a strong and durable tumor response for 21 (BLM) or 16 (BFS-1) days (Figure 5D-E), without side-effects and longer survival (Figure S4A-D). All other treatments, including DXR-SDDS (at an equivalent dose to IDA-SDDS) in HT treated mice, were ineffective (Figure 5D-E). Only when DXR-SDDS was administered in HT treated mice at a higher dose of 9 µmol/kg, a comparable tumor response was observed. Importantly, the absolute tumor uptake of DXR at a dose of 9 µm/kg was 25.2 ± 2.2 nmol/g, which is 1.6-times higher than that of IDA at dose of 2.7 µmol/kg (15.4 ± 3.5 nmol/g, *p* < 0.05), while comparable tumor responses were observed (Table 2), indicating a more potent antitumor activity of IDA, which correlates with observed *in vitro* cytotoxicity (Table 1). As expected, without hyperthermia administration of IDA-SDDS or DXR-SDDS was ineffective, indicating that triggered release is necessary. Histological examination of tumors after treatment thereafter confirmed necrosis of tumor cells when IDA-SDDS or DXR-SDDS were administered at maximum tolerated dose of 2.7 µmol/kg and 9 µmol/kg, respectively. In comparison with the control group, after 24 h of IDA- or DXR-SDDS with hyperthermia more cell death was observed, especially around vessels, which increased over time, eventually resulting in coagulative necrosis (Figure 5F).

## Discussion

Cancer is the leading cause of death in the Western World and gaining impact fast in developing countries 42, 43. Chemotherapy is one of the major pillars on which treatment relies 5. However, at present the biggest hurdle in cancer treatment is insufficient delivery of an active compound to solid tumors, regardless whether a chemotherapeutic, other small molecules, or an advanced pathway inhibitor is administered 4, 44, 45. This results in ineffective treatment as well as dose limiting side-effects. These side-effects not only prevent use of higher dosages but are often the reason to stop treatment. Therefore nano-scale delivery devices are used to improve drug concentration in tumor and diminish side-effects. The most clinically advanced DXR-SDDS, a lysolipid containing thermosensitive liposome, improved intratumoral DXR level 3.7-fold when heating was applied 46. Interestingly, in spite of the specific characteristics of these nano-devices and beneficial effects on pharmacokinetics, local concentration, intratumoral distribution and possible delivery sites, nano-devices are usually loaded with the drug of choice for the specific cancer from a classic oncological point-of-view 47. Here we propose to focus rather on the performance of a drug in association with the SDDS of choice. For instance, one potential, and currently unrealized benefit of SDDS is the ability to deliver drugs at therapeutic levels to tumors that are ineffective when delivered in free form due to an undesirable distribution and/or elimination profile. In this study, idarubicin, a drug that is used exclusively for the treatment of leukemia and myelodysplastic syndrome, was taken as an example. Due to the hydrophobicity IDA rapidly accumulates in circulating leukemic cells which are in direct contact with the injected drug, and this clearly benefits therapy. However, this fast sequestration of IDA by circulating cells limits delivery of the drug to tissue-embedded tumor cells, thus making IDA ineffective in most solid cancers when used in the free form. Both DXR and IDA have short blood circulation times of only minutes 34, 48. Encapsulation of IDA in SDDS not only prolongs circulation half-time in blood and reduces side-effects (Figure S5A-B). But more importantly, by using thermosensitive SDDS in combination with local hyperthermia IDA can be stably delivered to the target region and released locally, generating remarkably high drug concentrations available for tumor cells. An important aspect to consider is the short transit time of the SDDS in the tumor, which may be too short for to release DXR efficiently. We argue however that the more rapid release of idarubicin may result in 100% release when these SDDS pass through the heated tumor 33. However, fast release and high local concentrations over a short period of time may result in overloading of the tissue; cells are not able to sequester the drug fast enough. This may explain previous observations that dosing higher with heated-triggered SDDS containing DXR 49. Fast release of IDA however, together with the rapid extraction by tumor cells, can increase local accumulation over 100-fold in solid tumors, which thus yields a strong tumor response. A possibility to improve DXR delivery from SDDS is by prolonging heating time which indeed improves intratumoral concentration, but which may complicate treatment 50, 51.

**Figure 6:**
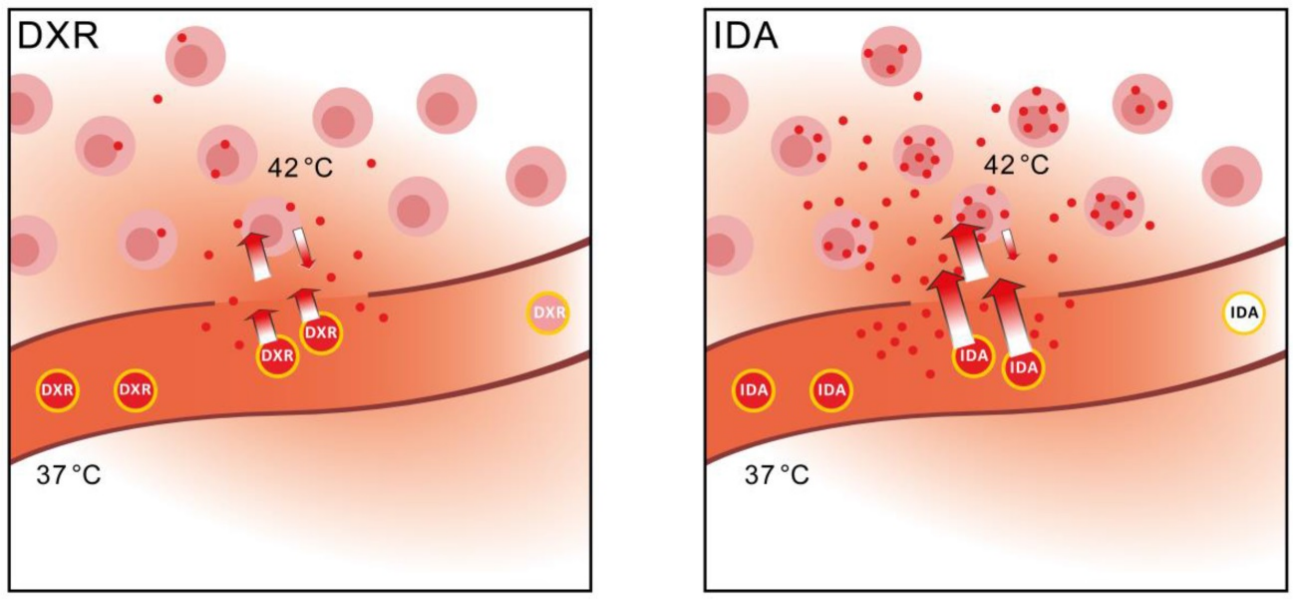
** Schematic overview of performance of IDA-SDDS in hyperthermia-treated mice versus DXR-SDDS.** When DXR-SDDS enter the heated region fast release occurs followed by uptake of doxorubicin by the tumor cells. However, uptake is limited, and retention not optimal, resulting in a relative high degree of doxorubicin leaving the tumor and re-entering the bloodstream. Moreover, release of content is not efficient enough and SDDS still containing drug leave the heated region resulting in inefficient drug delivery. When IDA-SDDS are entering the heated area ultra-fast release occurs and drug is simultaneously taken up by cells, accompanied by a high degree of retention in the cells. SDDS leaving the heated region are devoid of all idarubicin. This results in efficient delivery to the tumor cells with a steep decline when moving away from the tumor cells. However, overall the concentration of idarubicin is folds higher compared to doxorubicin.

Here we show that a change in thinking is needed and selection should be made for the optimal drug, considering both the optimal delivery method, i.e. particular SDDS, and cytotoxicity for targeted cells. DXR has been used most widely, largely because of the wide spectrum of activity, and ease of encapsulation. However, we postulate that drug uptake by tumor cells is the rate-limiting factor when SDDS are used in combination with locally triggered delivery. As the schematic overview illustrates in Figure 6, changing from DXR to a more hydrophobic anthracycline such as IDA, results in improved performance of the drug-SDDS combination with respect to tumor uptake efficiency and absolute uptake quantity (Table 2), and augments drug levels in tumor tissues. Hence, IDA, and similar drugs, may be promising candidates for treatment of solid tumors when encapsulated in thermosensitive SDDS. Moreover, use of smart drug delivery systems may open possibilities for drugs which may have been abandoned for poor performance or strong side-effects when used in the free form. As we demonstrated here, the fact that IDA is rapidly taken up by circulating cells becomes an advantage when encapsulated in SDDS. Our results show the importance of monitoring local delivery of a drug, detailed tracing of intratumoral distribution and determination of intracellular accumulation, and to relate that with clinical outcome. We envision that in a clinical trial, or for instance a trial with privately owned pets (dogs or cats with spontaneous tumors) current DXR-based SDDS are compared with IDA-SDDS, while above mentioned parameters are carefully recorded 52. Understanding how to choose the best drug for SDDS-mediated delivery is a timely question and may have a significant impact on the field by providing an answer to the hurdles currently faced.

## Conclusion

In summary, we developed a thermosensitive SDDS to encapsulate the antileukemia drug idarubicin, achieving an improved antitumor effect on solid tumors compared to doxorubicin, a reference drug used currently for most nano-carrier-mediated tumor therapies. Our results indicate that drugs similar to IDA can be loaded in stimuli-responsive materials formulated SDDS, in combination with local trigger delivery, not only to reduce side-effects on non-tumor tissue but also augments drug levels in the tumor. The hydrophobic nature of IDA could explain our observations, but different compounds have to be tested to show which characteristic of IDA determines the improved activity. The strategy proposed here also expands the potential application of therapeutics currently not considered because of undesirable characteristics when used in the free form. The novel thinking of selecting a drug with optimal kinetics for local delivery, and with a broad anticancer profile such as idarubicin, may have profound impact on clinical outcome.

## Supplementary Material

Supplementary figures and tables.

Supplementary IDA-SDDS video S1.

Supplementary DXR-SDDS video S2.

## Figures and Tables

**Table 1:** *In vitro* cytotoxicity of idarubicin and doxorubicin comparison. Data shown as IC_50_ value

	Co-incubation for 1 h			Co-incubation for 24 h		
	B16BL6	BLM	BFS-1	B16BL6	BLM	BFS-1
IDA#	0.048 *	0.036*	0.146 *	0.017*	0.010*	0.025*
DXR#	0.273	0.904	2.437	0.086	0.217	0.597

#IC_50_ (µM) of free idarubicin or doxorubicin; *Nonparametric Mann-Whitney test, *p* < 0.01.

**Table 2:** Uptake of idarubicin and doxorubicin in tumors when delivered by SDDS in combination with hyperthermia

	Uptake efficiency (%ID/g tumor)		Uptake absolute quantity (nmol/g tumor)	
	#Low dosage	High dosage	Low dosage	High dosage
IDA	13.6 ± 2.2*	13.0 ± 3.6*	15.4 ± 3.5*	43.8 ± 9.7*
DXR	4.5 ± 0.4	8.4 ± 0.7	4.2 ± 0.5	25.2 ± 2.2

# Low dosage: 2.7 µmol/kg; High dosage: 9 µmol/kg; IDA: idarubicin, DXR: doxorubicin; Data are presented as mean ± SD, N = 3; *p < 0.05.
