# Supplementary material for: Externally triggered smart drug delivery system encapsulating idarubicin shows superior kinetics and enhances tumoral drug uptake and response
Source: Theranostics. 2021 Mar 31;11(12):5700–12. doi: 10.7150/thno.55163 (PMC8058728; doi:10.7150/thno.55163)
Supplement: Supplementary file 1 — Supplementary figures and tables. [file thnov11p5700s1.pdf]

Supporting information

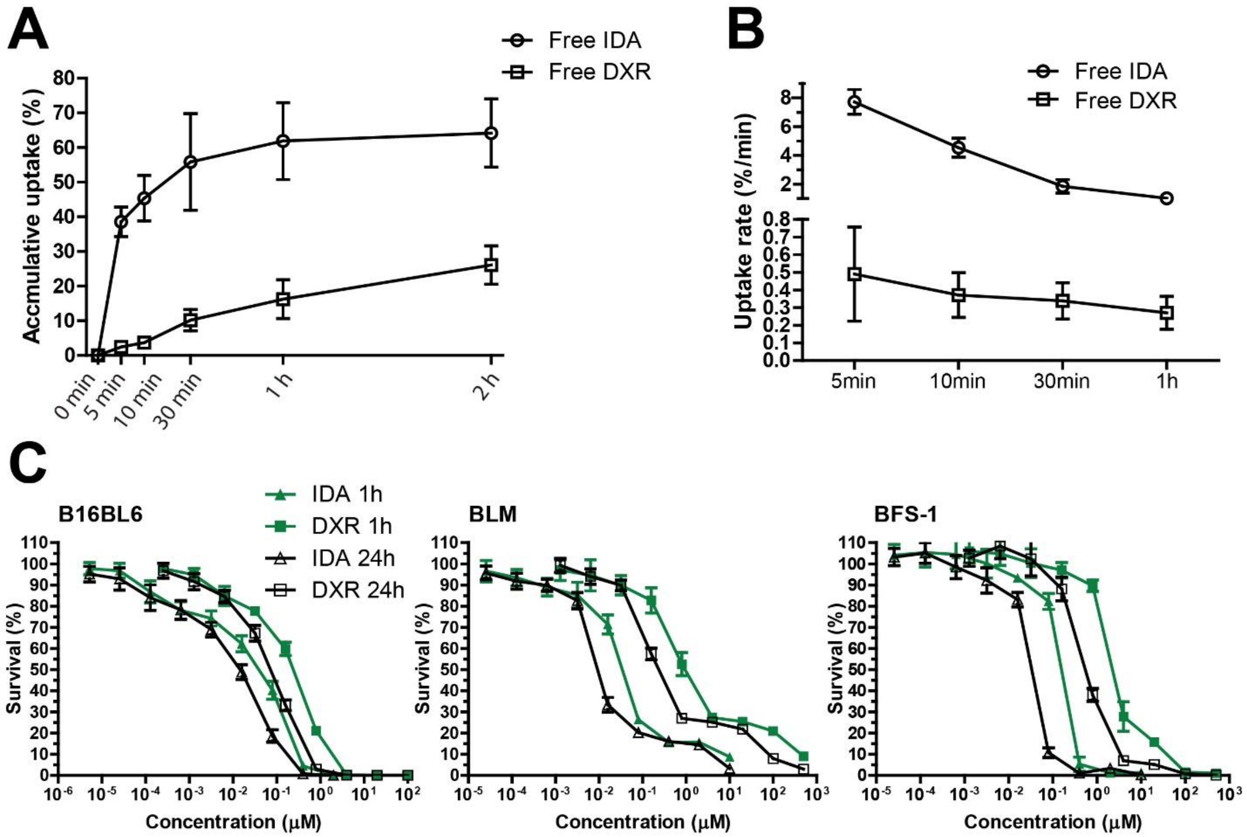

Supplementary figure 1. Faster cellular uptake and higher cytotoxicity of idarubicin (IDA) versus doxorubicin (DXR) *in vitro*. (A, B) Based on the average uptake percentage by the 3 cell lines shown in Figure 1A, calculations show a significantly higher cellular uptake rate for free IDA compared to free DXR (*n* = 4 per group). (C) B16BL6, BLM and BFS-1 cells were treated with either IDA or DXR for 1 or 24 h, showing a higher toxicity for IDA as compared to DXR (*n* = 3 per group). Data are represented as mean ± SD.

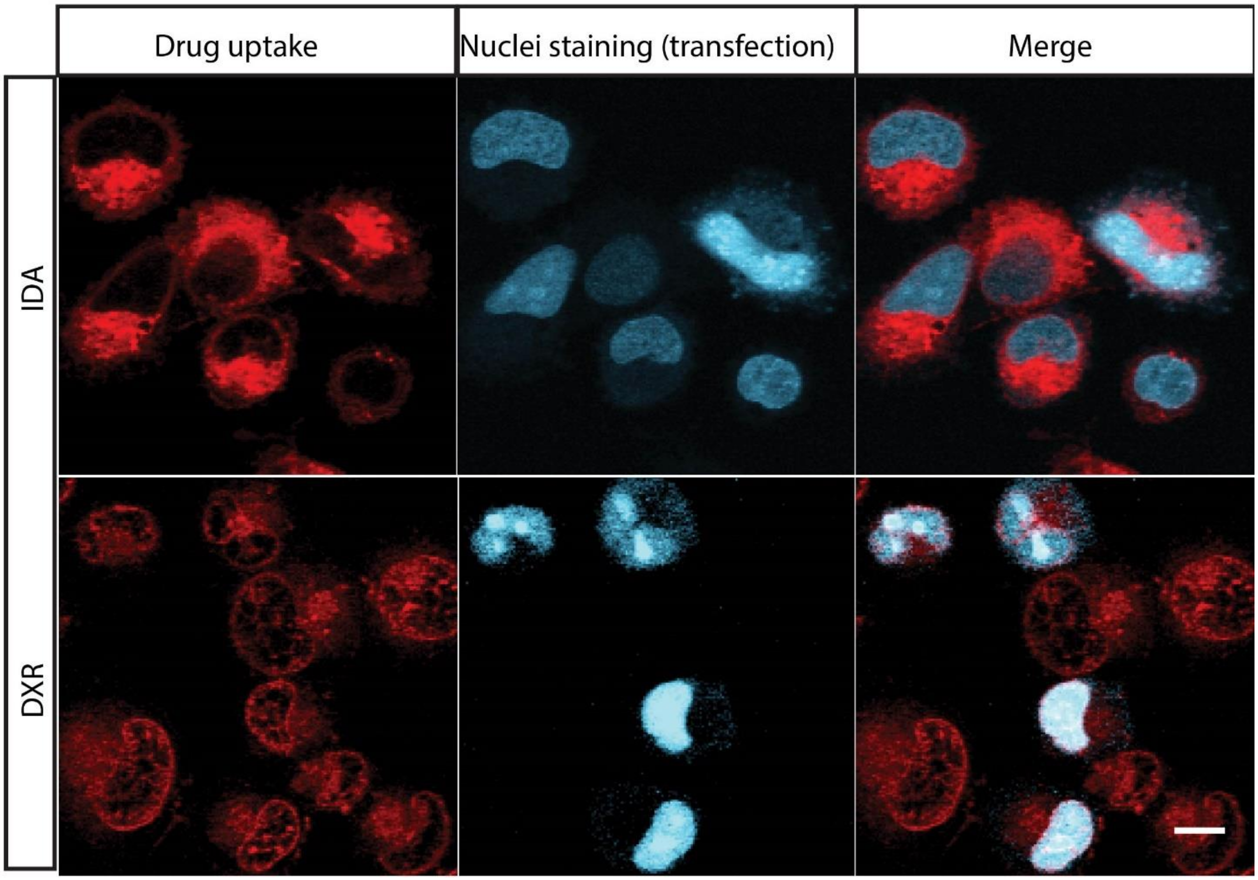

Supplementary figure 2. IDA (red) tends to accumulate in cytoplasm and little goes to the nucleus (blue) while DXR (red) accumulates predominantly in cell nucleus. Settings: IDA gain = 500, DXR gain = 630, resolution = 512 × 512. Scale bar = 10 µm. (Note, nuclei were stained through transient transfection resulting in not all cells to be positive).

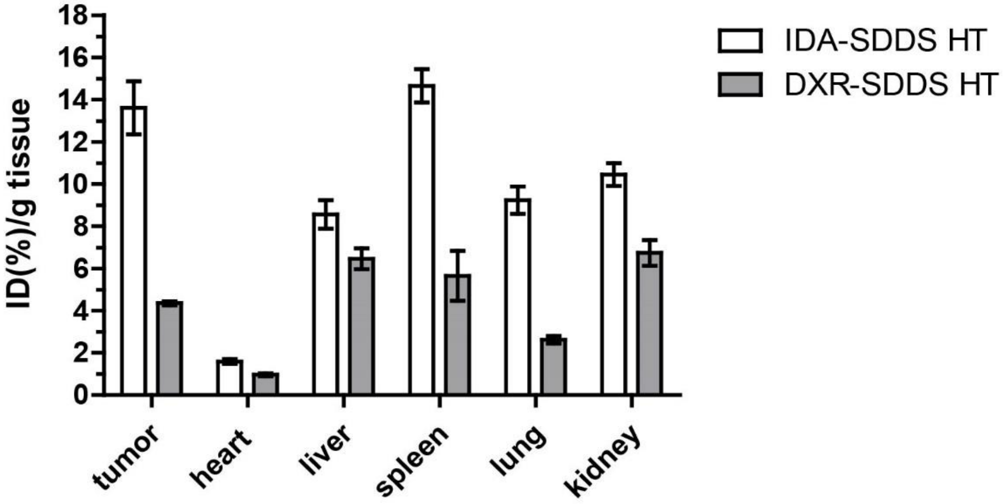

Supplementary figure 3. Biodistribution of IDA-SDDS and DXR-SDDS (2.7 μmol/kg) with HT. Data are represented as mean ± SEM, N = 3.

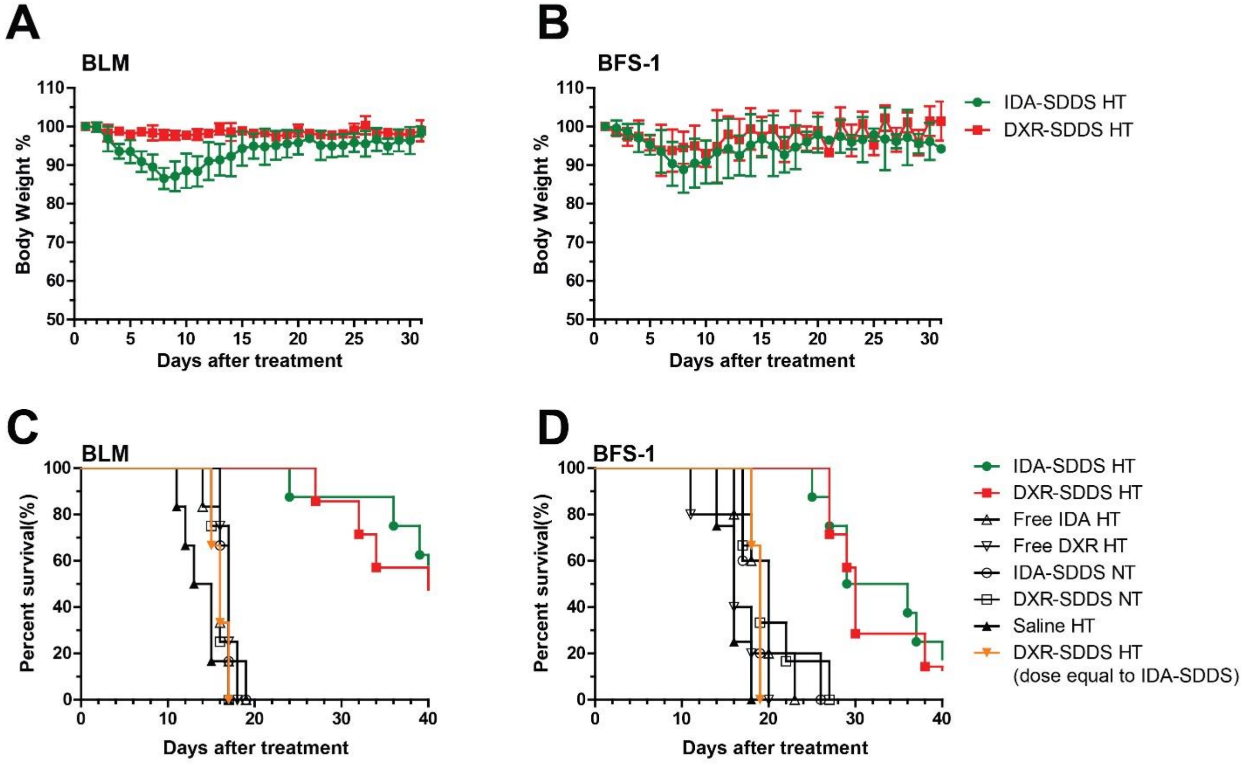

Supplementary figure 4. Administration of idarubicin (IDA)-SDDS (2.7 µmol/kg, green line) or doxorubicin (DXR)-SDDS (9 µmol/kg, red line) in combination with local hyperthermia (HT) inhibits tumor growth in both BLM and BFS-1 bearing mice, is accompanied by acceptable side-effects resulting in improved survival rates. (**A, B**) Body weight profiles of BLM (**A**) or BFS-1 (**B**) tumor-bearing mice after treatment with IDA- or DXR-SDDS plus HT. A reduction in body weight in the first week post treatment was observed in IDA-SDDS treated mice, followed by recovery. Mice treated with free drug with or without HT, or drug-containing SDDS combined with normothermia (NT) did not show significant weight loss (data not shown). Data are presented as mean ± SEM (n = 7 each group for IDA- or DXR-SDDS HT group, n = 5 each group for the rest). (**C, D**) Survival rates of BLM (**C**) or BFS-1 (**D**) tumor-bearing mice reveal longer survival period when treated with IDA- or high dose DXR-SDDS combined with HT as compared to the other groups. Mice we removed from the experiment when tumors reached a volume of 1500 mm^3. However, at the maximum tolerated dose no significant difference in survival rates was observed between mice treated with IDA- or high dose DXR-SDDS plus HT (n = 7 each group for IDA-/DXR-SDDS HT group, n = 5 each group for the rest).

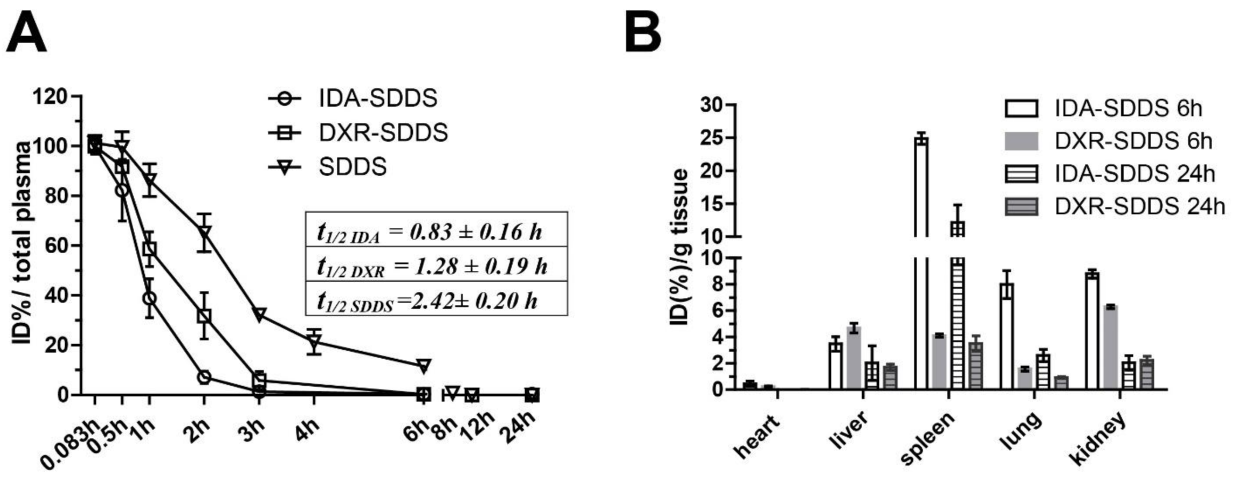

Supplementary figure 5. Pharmacokinetics and biodistribution of IDA- or DXR-SDDS comparison in healthy mice under normothermia. (A) Both IDA (2.7 µmol/kg) and DXR (9 µmol/kg) show prolonged circulation after encapsulation in SDDS (n = 9 mice per group), and (B) comparable biodistribution profiles (n = 3 mice per group). Data are represented as mean ± SEM.

Supplementary Table 1. Characterization parameters of IDA- and DXR-SDDS used in this study. Data are presented as mean ± SD, N = 4.

Liposome composition (mole)	Diameter (nm)		Polydispersity index (PDI)	
	Before	After	Before	After
IDA-SDDS (DPPC/DSPC/DSPE-PEG 6/3.5/0.5)	84 ± 2	81 ± 2	0.05 ± 0.02	0.04 ± 0.03
DXR-SDDS (DPPC/DSPC/DSPE-PEG 7/2.5/0.5)	86 ± 2	84 ± 3	0.03 ± 0.03	0.05 ± 0.02
